# Effects of Chitosan–PVA and Cu Nanoparticles on the Growth and Antioxidant Capacity of Tomato under Saline Stress

**DOI:** 10.3390/molecules23010178

**Published:** 2018-01-16

**Authors:** Hipólito Hernández-Hernández, Susana González-Morales, Adalberto Benavides-Mendoza, Hortensia Ortega-Ortiz, Gregorio Cadenas-Pliego, Antonio Juárez-Maldonado

**Affiliations:** 1 Departamento de Horticultura, Universidad Autónoma Agraria Antonio Narro, Saltillo 25315, Mexico; polo13_87-08@hotmail.com (H.H.-H.); abenmen@gmail.com (A.B.-M.); 2 Cátedras CONACyT, Departamento de Horticultura, Universidad Autónoma Agraria Antonio Narro, Saltillo 25315, Mexico; qfb_sgm@hotmail.com; 3 Centro de Investigación en Química Aplicada, Saltillo 25294, Mexico; hortensia.ortega@ciqa.edu.mx (H.O.-O.); gregorio.cadenas@ciqa.edu.mx (G.C.-P.); 4 Departamento de Botánica, Universidad Autónoma Agraria Antonio Narro, Saltillo 25315, Mexico

**Keywords:** antioxidant compounds, crop growth, enzymatic activity, NaCl stress

## Abstract

Chitosan is a natural polymer, which has been used in agriculture to stimulate crop growth. Furthermore, it has been used for the encapsulation of nanoparticles in order to obtain controlled release. In this work, the effect of chitosan–PVA and Cu nanoparticles (Cu NPs) absorbed on chitosan–PVA on growth, antioxidant capacity, mineral content, and saline stress in tomato plants was evaluated. The results show that treatments with chitosan–PVA increased tomato growth. Furthermore, chitosan–PVA increased the content of chlorophylls a and b, total chlorophylls, carotenoids, and superoxide dismutase. When chitosan–PVA was mixed with Cu NPs, the mechanism of enzymatic defense of tomato plants was activated. The chitosan–PVA and chitosan–PVA + Cu NPs increased the content of vitamin C and lycopene, respectively. The application of chitosan–PVA and Cu NPs might induce mechanisms of tolerance to salinity.

## 1. Introduction

Chitosan is a natural polymer derived from the deacetylation of chitin, which has great capacity in reticulation and cation exchange in acid solutions and has great affinity with metallic ions [1]. This polymer has been extensively investigated in agriculture and has been shown to stimulate growth and activate the defense mechanisms in plants [2]. Moreover, the use of nanotechnology is increasing in the medical sciences, electronics, pharmaceuticals, energy production, and even food additives. In agriculture, the main nanomaterials (NMs) being studied are composed of metals and metal oxides [3]. In this area, most of the research is focused on studying the effects on growth as well as the physiological and biochemical changes of plants [3,4]. In general, it is reported that, most of the time, concentrations of NMs less than 100 ppm applied via soil, foliar, or seeds, especially 10–40 mg kg^−1^, have been shown to have beneficial effects on plant growth, while higher concentrations have inhibitory effects. However, these effects depend on factors such as the properties of NMs, plant species, soil dynamics, and soil microbial communities, among others [5]. Copper nanoparticles (Cu NPs) are one of the NMs based on metals, which have been shown to improve plant growth, increase chlorophyll concentration, and increase the concentration of phenolic compounds and defensive enzymes (catalase (CAT), superoxide dismutase (SOD) and phenylalanine ammonium lyase (PAL) among others) when applied at low concentrations (0.05–1.0 mg L^−1^) in seeds or soil [6,7,8,9]. However, one of the disadvantages of NPs in general is their insolubility in water, which limits the evaluation of their toxicity in experiments. Therefore, new alternatives are being sought, such as the encapsulation of the nanoparticles in order to achieve a controlled release. With respect to this, it has been reported that encapsulated Cu NPs are less toxic than free Cu NPs and even copper sulfate [6,10,11]. A polymer that is being used for the encapsulation of NPs is chitosan (Cs) due to its biocompatibility, biodegradability, non-toxicity, and antibacterial and adsorption abilities [12,13,14]. Some investigations based on Cu NPs encapsulated in chitosan demonstrate that they can increase the growth of plants and the amount of lycopene in tomato fruits, induce defensive enzymes in plants, such as catalase, peroxidase, superoxide dismutase, and phenylalanine ammonium lyase, and act as an antifungal agent against various phytopathogenic fungi [7,8,11].

On the other hand, it is known that saline stress affects a wide variety of crops worldwide, and it is reported that over 6% of the world’s land is affected by salinity [15]. Some of the effects of saline stress on plants include a reduction in the rate of expansion of the foliar surface; a more negative nature of the water and osmotic potential; an increase in the thickness of the epidermis and the mesophyll; an increase in the levels of Na and Cl; a decrease in Ca, K, and Mg levels; and induction of the activity of certain antioxidant enzymes, such as catalase, peroxidase, glutathione reductase, and superoxide dismutase [16]. There are studies on the effect of NMs based on metals in plants subjected to saline stress. Some of them report that NPs of ZnO in concentrations of 15–30 mg L^−1^ have positive responses in the metabolism of tomato plants under salt stress [17]. Likewise, it has been shown that concentrations of 0.05–2.5 mg L^−1^ of Ag NPs can improve the tolerance of tomato plants to salinity [18]. In canola, it was reported that concentrations of 200 and 1000 mg kg^−1^ of CeO_2_ NPs improved the growth and physiology of plants under saline stress, but did not completely alleviate it [19]. Considering the importance of research examining chitosan and NM in different plants, the following study was carried out to evaluate the effect of chitosan–PVA and Cu NPs on growth, antioxidant capacity, and mineral content in tomato plants under conditions of saline stress.

## 2. Results and Discussion

### 2.1. Growth and Development of Tomato Plants

For the growth and development parameters evaluated in this study, we showed statistical differences between treatments (Table 1). In plant height, the absolute control was significantly different to those that underwent treatments with chitosan–PVA. Stem diameter was 6.5% larger with chitosan–PVA + Cu NPs compared to the absolute control. For the number of leaves, those treated with chitosan–PVA + Cu NPs had 3.3% smaller leaves compared to the absolute control. However, the same treatment shows a larger number of fruits (20%) compared to the absolute control without saline stress. The fresh and dry weight of the roots after being treated with chitosan–PVA were significantly larger than the absolute control. These results indicate a stimulatory effect of chitosan–PVA on root growth. Under saline stress, there were no differences between treatments for these variables. Under saline stress, only two variables have differences between treatments. The plant height was better with chitosan–PVA, although this was not different from the NaCl treatment. The yield was 10.6% better with chitosan–PVA + Cu NPs compared to only chitosan–PVA.

It has been previously reported that Cu NPs encapsulated in chitosan increase the fresh and dry weight as well as the number of clusters and fruits per tomato plant [6,7]. Previously, Pinedo-Guerrero et al. [9] reported that Cu NPs in Cs–PVA hydrogels decrease the height of pepper plants. Therefore, plants treated with Cu NPs in Cs–PVA hydrogels decrease in height, which could be due to the generation of reactive oxygen species (ROS). However, only chitosan–PVA had beneficial effects on this variable under saline stress. Regarding fruit yield per plant, there were only significant differences between treatments with saline stress compared to those without stress. As expected, saline stress negatively affected the yield of the plants. This type of stress generally has negative effects by reducing crop growth and yield [16], as it was demonstrated in this study. The results of this work show that Cu NPs have a positive effect on fruit yield under saline stress conditions. CeO_2_ NPs have increased total biomass in canola plants exposed to salinity, but they have not fully compensated for the negative effects of saline stress [19] as observed here.

### 2.2. Changes in Leaf Pigments

The results show that saline stress increased the content of chlorophyll a, chlorophyll b, total chlorophyll, and carotenoids compared to conditions without stress (Table 2). These increases could be used as a biochemical indicator for tolerance to salinity [20]. Without stress conditions, only chlorophyll a/b ratio was affected by treatments. Chitosan–PVA + Cu NPs was 4.3% smaller than the absolute control.

Under saline stress conditions, chitosan–PVA increased chlorophyll a and b content (15 and 22%, respectively), total chlorophyll (16%), and carotenoids (15%) compared to NaCl treatment (Table 2). Ma et al. [21] reported that chitosan increased the chlorophyll content in wheat plants under conditions of saline stress. Thus, chitosan could help minimize the effect of saline stress on tomato plants. In addition, the chlorophyll a/b ratio was significantly higher in the control compared to the rest of the treatments. The increase in the chlorophyll a/b ratio is associated with a change in the pigment composition of the photosynthetic apparatus towards a solar-type chloroplast, which has less chlorophyll light-harvesting proteins (LHCPs) [22]. As a result, treatment with chitosan and saline stress increased the amount of LHCPs in the leaves as a protection mechanism.

### 2.3. Changes in Enzymatic Activity

The results without saline stress show that the activity of the enzymes ascorbate peroxidase, catalase, superoxide dismutase, glutathione peroxidase, and phenylalanine ammonium lyase was increased in tomato leaves after chitosan–PVA + Cu NPs treatment (57%, 111%, 92%, 39%, and 62% respectively) compared to the absolute control (Table 3). Thus, it is shown that Cu NPs produce positive stress in the plants by activating their defense mechanism [9]. In a previous study, it was shown that Cu NPs in chitosan–PVA hydrogels increase the CAT activity in tomato leaves, whereas ascorbate peroxidase (APX), glutathione peroxidase (GPX), and SOD did not show significant differences [7]. This may be due to the fact that a lower concentration of Cu NPs was applied. In mung bean leaves, it was reported that Cu NPs encapsulated in PEG increased the activity of CAT and SOD enzymes [6]. In another study evaluating CeO_2_ NPs coated with citric acid, the activity of the CAT enzyme was increased in tomato leaves [23]. On the other hand, phenylalanine ammonium lyase is an enzyme of great importance as it is key in the synthesis of metabolites of defense against pathogens. Some authors have stated that Cu NPs coated with chitosan help protect plants from pathogens such as *Alternaria* and *Fusarium* [11]. Thus, Cu NPs in chitosan–PVA hydrogels could function as inducers of systemic resistance in plants. Under saline conditions, differences between treatments were demonstrated in superoxide dismutase only. The chitosan–PVA was 31% larger that the NaCl treatment.

In fruits without saline stress, chitosan–PVA + Cu NPs increased catalase (122%) and superoxide dismutase (40%) compared to absolute control. While GPX activity was increased by chitosan–PVA (32%) and chitosan–PVA + Cu NPs (29%). Regarding this, Cu NPs can function as elicitors in the activation of defensive enzymes in plants [9], helping them cope with certain types of biotic and abiotic stress. In a previous study, the Cu NPs and chitosan did not increase the activity of these enzymes, which could be because the concentration used was much lower [7]. However, under saline stress, CAT and SOD was larger in NaCl treatment. This is because saline stress increases ROS production and therefore the plant activates its antioxidant defense mechanism [24].

### 2.4. Changes in Antioxidant Capacity

The results show that, without saline stress, chitosan–PVA + Cu NPs presented higher reduced glutathione (GSH) (58%) in the leaves compared to the absolute control (Table 4). GSH is critical in the glutathione–ascorbate cycle and is crucial for biotic and abiotic stress, as it induces defense responses against pathogens, such as *Pseudomonas syringae* and *Phytophthora brassicae* [25]. Thus, Cu NPs could be used as the elicitor to produce this compound. The total content of phenols was increased with both chitosan–PVA and chitosan–PVA + Cu NPs treatments (60 and 53%, respectively). Total phenols are antioxidants that trigger a series of secondary metabolites synthesized through the pathway of shikimic acid or malonic acid, which exert cellular signaling functions under conditions of abiotic stress [26]. Since the chitosan–PVA increases phenolic compounds in plants, it might induce tolerance to salt stress.

Under saline stress conditions, a reduction of the antioxidant capacity by ABTS (2,2′-azino-bis [3-ethylbenzothiazoline-6-sulphonic acid]) was observed in both chitosan–PVA and chitosan–PVA + Cu NPs treatments compared to the NaCl treatment. The ABTS technique quantifies the hydrophilic compounds [27], so it can be said that chitosan–PVA affected this type of compound under saline stress.

In tomato fruits without saline stress, the antioxidant capacity DPPH (2,2-Diphenyl-1-Picrylhydrazyl) was larger with chitosan–PVA + Cu NPs, which were both equivalent to Trolox and equivalent to vitamin C, compared to the absolute control (26% and 29%, respectively) (Table 5). The reduced glutathione was increased with chitosan–PVA by 32%. There was more vitamin C after both chitosan–PVA and chitosan–PVA + Cu NPs treatments, with increases of 47% and 41%, respectively, compared to the absolute control. The content of lycopene in tomato fruits increased significantly with chitosan–PVA + Cu NPs (77%).

Under saline stress, the antioxidant capacity DPPH was increased with chitosan–PVA by 19% and 21%, which is equivalent to Trolox and equivalent to vitamin C, respectively. The reduced glutathione was increased in NaCl treatment. Similar to the situation without stress, Vitamin C was increased with both chitosan–PVA and chitosan–PVA + Cu NPs treatments (42% and 34%, respectively). Similar to the situation without stress, the content of lycopene was increased with chitosan–PVA + Cu NPs by 42% compared to NaCl treatment. These results confirm that chitosan–PVA positively affects vitamin C, while chitosan–PVA + Cu NPs positively affect the lycopene on tomato fruits. There are several reports on the effective application of chitosan as a coating for fruits to reduce the loss of antioxidants, such as ascorbic acid, anthocyanins, and total polyphenols [28]. This is of great importance since humans must ingest vitamin C through sources, such as fruits, because the human body does not possess the enzymatic capacity to produce it [29]. On the other hand, it has previously been shown that Cu NPs in Cs–PVA hydrogels increase the lycopene content in tomatoes [7]. It has also been shown that the application of NPs of ZnO and TiO_2_ in foliar form and into the soil increased the content of lycopene in tomatoes [30]. Lycopene is an antioxidant that protects human cells from oxidative stress, which are produced by free radicals that are a larger cause of cardiovascular disease, cancer, and premature aging [31]. Therefore, chitosan and chitosan–PVA + Cu NPs could be an alternative for increasing this type of antioxidants in tomato fruits.

### 2.5. Mineral Content on Leaves and Fruits

Without saline stress, the treatments affect the K, Ca, and Cu content in leaves (Table 6). The K and Ca content was reduced after treatment with chitosan–PVA, compared to the absolute control (14% and 8% respectively). The Cu content was reduced with both chitosan–PVA and chitosan–PVA + Cu NPs by 7%. These results are consistent with Juarez-Maldonado et al. [7], who showed that chitosan and Cu NPs decrease the Cu content in tomato plants.

Under saline stress conditions, chitosan–PVA + Cu NPs significantly increased N content (7%) compared to the NaCl treatment. It has also been reported that saline stress affects the N content in canola leaves [32]. Moreover, it had already been confirmed that Cu NPs are involved in nitrogen metabolism by increasing the activities of the enzymes nitrate reductase, nitrite reductase, glutamine synthase, and glutamate synthase [6]. This could explain the increase in N content in leaves. The content of Na was larger with NaCl treatment. Both chitosan–PVA and chitosan–PVA + Cu NPs decreased the content of Na (52% and 38%, respectively). This could promote the tolerance against salt stress, because the sodium itself causes toxicity in cells and alters cellular homeostasis, which causes osmotic stress and ionic toxicity that affects plant growth [33]. Some authors have shown that CeO_2_ NPs modify the formation of apoplectic barriers, which improves tolerance to salt stress in *Brassica napus* plants [32]. In the present study, the content of Fe was diminished with both chitosan–PVA and chitosan–PVA + Cu NPs (17% and 12%, respectively).

In tomato fruits without saline stress, chitosan–PVA + Cu NPs increased N content (15%) compared to the control, which was an effect similar to that in the leaves. The content of Mg was reduced by these same treatments. This could be associated with an increase of chlorophyll in the leaves. Under saline stress conditions, no differences were shown for mineral content. It is known that Ca and K levels decrease due to salinity [16], although this was not observed in this present study.

## 3. Materials and Methods 

### 3.1. Materials

Cu NPs (spherical morphology, 99.8% purity and average diameter of 25 nm) were purchased from SkySpring Nanomaterials, Inc. Houston, TX, USA. Chitosan (*Mv* = 200,000 g/mol) was obtained from Marine Chemicals, New Delhi, India. Polyvinyl alcohol (*Mw* = 30,000–50,000; hydrolysis 98%), l-ascorbic acid, polyvinylpyrrolidone, bovine serum albumin, Bradford reagent, standard glutathione, Folin–Ciocalteu, 5,5-dithio-bis-2-nitro benzoic acid, and l-Phenylalanine were obtained from Sigma Aldrich, St. Louis, MO, USA. The amount of SOD (U/mL) was determined with a commercial Cayman Chemicals kit. ABTS or 2,2′-azino-bis (3-ethylbenzothiazoline-6-sulphonic acid) and potassium persulfate were obtained from Sigma Aldrich, St. Louis, MO, USA. The DPPH (2,2-diphenyl-1-Picrylhydrazyl) used was from Cayman Chemicals. Tomato seeds (hybrid var. “Huno F1” saladette type and indeterminate growth) were purchased from Harris Moran Seed Company (Modesto, CA, USA).

### 3.2. Synthesis of Chitosan-Polyvinyl Alcohol Hydrogels (Cs–PVA) and Absorption of Cu NPs

This was carried out according to the methodology of Pinedo-Guerrero et al. [9]. Experiments were carried out in the pilot plant of the Applied Chemistry Research Center (CIQA) according to the following methodology. Two hundred fifty milliliters of 2% *w/v* chitosan in 1% *v/v* acetic acid and 250 mL of 4% *w/v* polyvinyl alcohol (PVA) were first dissolved by mixing for 2 h at 300 rpm and 60 °C to obtain a complex in a 1:2 ratio (Cs/PVA). The pH of the Cs–PVA was 4.33. Subsequently, 2.27 mL of the crosslinker (glutaraldehyde, molar ratio R = [–CHO]/[–NH_2_] = 0.5) were added at 450 rpm for 5 min at 25 °C, and 100 mL of 6% *w/v* NaOH were then added at 300 rpm, which was maintained at 25 °C for 1 h to obtain a Cs–PVA hydrogel. The Cs–PVA hydrogels were washed twice with distilled water at 50 °C, purified once with ethanol, and finally dried for 24 h at 60 °C in an oven until a constant weight was achieved. After this, 100 mg of the Cu NPs were dispersed in a 1% Tween solution using an ultrasound for 5 min (50-watt power and 70% frequency), and a dilution was then prepared to obtain a concentration of 10 mg. These were subsequently absorbed in 1 g of Cs–PVA hydrogel and dried at a temperature of 60 °C for 24 h to obtain a constant weight.

### 3.3. Experimental Development and Growth Conditions

Tomato plants (*Solanum lycopersicum* L.) were established in a multi-tunnel greenhouse with polyethylene cover of the Department of Horticulture of the Autonomous University of Agriculture Antonio Narro. The average temperature was 21 °C, while there was an active photosynthetic radiation of 565 μmol m^−2^ s^−1^ and an average relative humidity of 51%. The planting density was three plants per square meter. A soilless system culture was used, and the substrate used was a mixture of peat moss and perlite (1:1) placed in bags of black polyethylene with 12 L capacity. The plants were maintained with a single stem by pruning (removing the axillary buds). Plant growth was limited to 70 days after transplanting, eliminating apical growth. The experiment was carried out in two ways: (1) an evaluation of the effect of Cs–PVA hydrogel treatment (1 g per plant), with 10 mg of Cu NPs absorbed on 1 g of Cs–PVA hydrogel and the absolute control; and (2) a valuation of the same treatments with an application of 100 mM NaCl in the nutrient solution from the third week after transplantation throughout the entire duration of experimentation. For the application of the treatments, 1 g of Cs–PVA hydrogel was distributed in the lower, middle, and upper parts of the pot before the transplant to obtain a better dispersion of the different hydrogels in the substrate and in the root area of the plant. A directed irrigation system was installed, and four irrigations per day at different times were applied. The amount of water applied was different for each phenological stage, applying close to 2.5 L per plant per day in the stages with higher consumption. The nutrient solution Steiner [34] was used with the following micronutrients in chelated form using EDTA (2,2′,2″,2′″-[Ethane-1,2-diyldinitrilo] tetraacetic acid)/Fe EDTA = 3.75 ppm; Mn EDTA = 1.85 ppm; B = 0.35 ppm; Zn EDTA = 0.30 ppm; Cu EDTA = 0.15 ppm; Mo = 0.10 ppm. The nutrient solution was applied in different concentrations to provide the necessary nutrients to tomato plants. The concentration applied was 25% for the first two weeks after the transplantation, 50% for the third and fourth weeks, 75% for the fifth week, and 100% for the rest of the crop cycle. 

### 3.4. Tomato Growth Parameters

Sampling of plant height and stem diameter was performed 75 days after transplantation. At this time, the growth apex of plants was eliminated. Plant height was measured with a flexometer from the surface of the substrate to growth apex. The stem diameter was measured with a digital caliper between the first and second leaves of the plant base. At 170 days after transplantation, the yield by plant was determined. The yield considers the weight of all fruits harvested throughout the time of experimentation. The number of fruits harvested was also obtained. Moreover, the plants were cut on the surface of the substrate, and the fresh weight of the roots and shoots (stem and leaves) was then measured. The dry weight of the roots and shoots was obtained after they were dried in a drying oven (Drying Oven model DHG9240A) for 72 h at a constant temperature of 80 °C.

### 3.5. Determination of Chlorophyll and Carotenoid Content

The extraction methodology was performed according to Pocock et al. [35] with modifications. The tomato leaves of each treatment were cryogenized with liquid nitrogen. Subsequently, 0.1 g were taken and 1 mL of 100% acetone was added. This mixture was centrifuged at 3024× *g* for 5 min. Subsequently, 0.05 mL of the supernatant were taken and 0.95 mL of 80% acetone (2.5 mM sodium phosphate buffer pH 7.8) were added. A blank composite mixture of 80% acetone was used. The content of chlorophyll a (Chl a) and chlorophyll b (Chl b) was determined by spectrophotometry using the absorbance (Abs) read at 664 nm and 647 nm in Equations (1) and (2) according to Porra [36]. Total chlorophyll is the sum of Chl a and Chl b. Total carotenoids (TC) were determined using the absorbance read at 470 nm and data of Chl a and Chl b in Equation (3) according to Wellburn [37]. All results were expressed in mg g^−1^ fresh weight.

(1) $$\mathrm{Chl}\;a=12.25\times {\mathrm{Abs}}_{664}-2.55\times {\;\mathrm{Abs}}_{647}$$

(2) $$\mathrm{Chl}\;b=20.31\times {\;\mathrm{Abs}}_{647}-4.91\times {\;\mathrm{Abs}}_{664}$$

(3) $$\mathrm{TC}=\left(1000\times {\;\mathrm{Abs}}_{470}-1.82\times \mathrm{Chl}\;a-\;85.02\times \mathrm{Chl}\;b\right)/198$$

### 3.6. Extraction of Biomolecules

After 60 days of transplantation, random plants were selected, and the third fully expanded young leaf was taken for biochemical analysis. The fruits were selected at random after a harvest to verify that they were not physically damaged, uniform, and in the 6th stage of maturity (light red) according to the visual color pattern used by the United States Department of Agriculture [38]. Samples were stored at −80 °C until use. For the enzymatic and non-enzymatic determination, 200 mg of lyophilized fruits and cryogenized leaves with liquid nitrogen of each treatment and 20 mg of polyvinylpyrrolidone were weighed. After this, 1.5 mL of phosphate buffer with a pH of 7–7.2 (0.1 M) were added, and the mixture was then subjected to micro-centrifugation at 12,000 rpm for 10 min at 4 °C. The supernatant was filtered with a nylon membrane [39]. Dilutions of the extract were prepared in a ratio of 1:20 with the phosphate buffer. Using this extract, we determined proteins, catalase, ascorbate peroxidase, superoxide dismutase, glutathione peroxidase, phenylalanine ammonium lyase, reduced glutathione, ABTS, and DPPH antioxidant capacity.

### 3.7. Proteins

Protein quantification was determined by the Bradford [40] method, which involves taking 20 μL of the extract or standard and adding 980 μL of the Bradford reagent. After 5 min, the absorbance was read at 595 nm on a UV-Vis spectrophotometer (Thermo Scientific Model G10S, Waltham, MA, USA). A calibration curve was created with standard bovine serum albumin (0.02–0.1 mg mL^−1^) (Figure S1).

### 3.8. Catalase (EQ 1.11.1.6)

We quantified catalase levels according to Beers and Sizer [41]. In this process, 70 μL of the extract were taken, 920 μL of phosphate buffer were added, and 10 μL of H_2_O_2_ (2 M) were then added to initiate the reaction. A blank was used for each sample, which contained 930 μL of phosphate buffer and 70 μL of extract. The absorbance was recorded initially and then every 20 s for 3 min. The decomposition of H_2_O_2_ was followed by the decrease in absorbance at 270 nm in a UV-Vis spectrophotometer (Thermo Scientific Model G10S, Waltham, MA, USA). A calibration curve was created with H_2_O_2_ (20–100 mM) and the results were expressed as mM H_2_O_2_ min^−1^ per total protein (mg g^−1^) (Figure S2).

### 3.9. Ascorbate Peroxidase (EQ. 1.11.1.1)

This was determined according to Nakano and Asada [42]. In 100 μL of the extract, 600 μL of phosphate buffer were added to 100 μL of EDTA (1 mM), 100 μL of ascorbate (5 mM), and 100 μL of H_2_O_2_ (1 mM) to initiate the reaction. A blank was used with 700 μL of phosphate buffer, 100 μL of EDTA (1 mM), 100 μL of ascorbate (5 mM), and 100 μL of H_2_O_2_ (1 mM). The oxidation of ascorbate was estimated by the decrease in absorbance at 266 nm after 1 min on a UV-Vis spectrophotometer. A calibration curve was prepared with ascorbate (10–100 μmol), and the results were expressed in μmol of ascorbate min^−1^ by total proteins (mg g^−1^) (Figure S3).

### 3.10. Superoxide Dismutase (EQ 1.15.1.1)

For this analysis, 200 μL of the radical detector (tetrazolium salt) were added to 10 μL of the extract or standard. To initiate the reaction, 20 μL of xanthine oxidase were added. This was then incubated for 30 min at room temperature, and the absorbance at 450 nm was then read in a microplate reader ELISA (BioTek model ELx-808 IU, Winooski, VT, USA). A calibration curve was prepared with standard SOD (0–0.05 U mL^−1^), and the results were expressed as U mg^−1^ protein (Figure S4).

### 3.11. Glutathione Peroxidase (EQ 1.11.1.9)

This was conducted according to the modified Flohé and Günzler [43] technique, using H_2_O_2_ as a substrate. For the enzymatic reaction, 0.2 mL of the extract were placed in an Eppendorf tube with 0.4 mL of 0.1 mM reduced glutathione and 0.2 mL of 0.067 M Na_2_HPO_4_. For the non-enzymatic reaction, the previous reagents were used without the extract. These mixtures were preheated in a water bath at 25 °C for 5 min, and 0.2 mL of 1.3 mM H_2_O_2_ were then added to initiate the catalytic reaction. This mixture was allowed to react for 10 min and stopped by the addition of 1 mL of 1% trichloroacetic acid. This reaction mixture was placed in an ice bath for 30 min. The mixture was then centrifuged at 3000 rpm for 10 min, and 0.24 mL of the supernatant or standard were then taken. After this, 1.1 mL of 0.32 M Na_2_HPO_4_ and 0.16 mL of 1 mM of the dithio-5-dithio-2-nitro benzoic acid (DTNB) dye were added. A blank was used, which contained 1.1 mL of 0.32 M Na_2_HPO_4_, 0.16 mL of 1 mM DTNB, and 0.24 mL of phosphate buffer. Subsequently, the assay was read at an absorbance at 412 nm on a UV-Vis spectrophotometer [44]. The enzymatic activity was calculated as a decrease of GSH within the reaction time as compared to the non-enzymatic reaction. A calibration curve with standard reduced glutathione (20–100 μM) was created and the results were expressed in μmol of glutathione per min^−1^ by total proteins (mg g^−1^) (Figure S5).

### 3.12. Phenylalanine Ammonium Lyase (EQ 4.3.1.5)

This was determined according to Sykłowska-Baranek et al. [45] with modifications. A total of 0.1 mL of the enzymatic extract were taken, and 0.9 mL of l-phenylalanine (6 mM) were added. After 30 min of incubation at 40 °C, the reaction was stopped with 0.25 mL of 5 N HCl. The samples were placed in an ice bath, and 5 mL of distilled water were added. The absorbance was determined at 290 nm on a UV-Vis spectrophotometer. A calibration curve was prepared with cinnamic acid (300–3000 μmol), and the results were expressed as the production of 1 mM cinnamic acid per min by total proteins (mg g^−1^) (Figure S6).

### 3.13. Reduced Glutathione (GSH)

It was performed calorimetrically by reaction with DTNB. In an Eppendorf tube, 0.48 mL of the extract were placed, and 2.2 mL of 0.32 M Na_2_HPO_4_ and 0.32 mL of the 1 mM DTNB dye were then added. After completely mixing, the absorbance at 412 nm was read in a UV-Vis spectrophotometer [44]. The data obtained were interpolated to a calibration curve previously standardized with GSH, and the results were expressed in μmol of GSH per mg of total proteins.

### 3.14. ABTS (2,20-Azinobis-3-ethylbenzotiazoline-6-sulphonic Acid)

The ABTS technique measures the hydrophilic and lipophilic compounds, and this was performed according to the methodology of Miller et al. [46]. The ABTS cation was generated through the interaction of 19.2 mg of ABTS dissolved in 5 mL of HPLC grade H_2_O with 88 μL of potassium persulfate at a concentration of 37.8 mg mL^−1^. The cation was incubated in the dark and at room temperature for 16 h. The activated ABTS radical was diluted with ethanol to an absorbance of 0.7 ± 0.02 at 734 nm using a UV-Vis spectrophotometer [47]. Subsequently, 5 μL of the extract or standard were taken, and 395 μL of the diluted ABTS solution were added. After 6 min, the absorbance was recorded. Ethanol was used as a blank. Two calibration curves were made: with Trolox (0–1 mM) and standard ascorbic acid (0–0.25 mg mL^−1^). The results were expressed as Trolox equivalents in mM 100 g^−1^ fresh weight (leaf) and dry (fruit) and ascorbic acid equivalents in mg 100 g^−1^ fresh weight (leaf) and dry (fruit) weight (Figures S7 and S8).

### 3.15. DPPH (2,2-Diphenyl-1-picrylhydrazyl)

The DPPH technique measures the hydrophilic and lipophilic compounds. This was performed according to the Brand-Williams et al. [48] methodology with some modifications. The stock solution was prepared by mixing 2.5 mg of the DPPH radical with 100 mL of methanol. The absorbance of the solution was adjusted to 0.7 ± 0.02 at 515 nm using a UV-Vis spectrophotometer. After this, 10 μL of extract or standard were taken, and 390 μL of the diluted DPPH radical were added. Methanol was used as a blank. The decrease in absorbance at 515 nm was measured after 30 min. Two calibration curves were made: with Trolox (0–1 mM) and standard ascorbic acid (0–0.12 mg mL^−1^). The results were expressed as Trolox equivalents in mM·100 g^−1^ fresh weight (leaf) and dry (fruit) and ascorbic acid equivalents in mg 100 g^−1^ fresh weight (leaf) and dry (fruit) weight (Figures S9 and S10).

### 3.16. Total Phenols

This was determined according to the methodology of Singleton et al. [49] with modifications. After 250 mg of cryogenized sheet with liquid nitrogen was taken, 1 mL of 80% methanol was added, and this mixture was then centrifuged at 10,000 rpm for 15 min. The supernatant was recovered, and the same procedure was performed with concentrated methanol for the pellet. The supernatant was adjusted to 2 mL with concentrated methanol and placed in the dark. Subsequently, 200 μL of the extract and 1.5 mL of distilled water were added, and 100 μL of Folin–Ciocalteu reagent were then added. After this, 200 μL 20% NaCO_3_ were added, and the mixture was allowed to rest for 30 min. The absorbance was read at 765 nm on a UV-Vis spectrophotometer. A calibration curve was prepared with gallic acid (0.02–0.4 mg mL^−1^), and the results were expressed as mg of gallic acid per gram of fresh weight (Figure S11).

### 3.17. Lycopene

The content of lycopene was determined according to Fish et al. [50]. A total of 3 mL of phosphate buffer solution (pH of 7) were added to 3 g of pericarp of fresh fruit and ground in a mortar. Subsequently, 2 mL of the sample were taken, and 4 mL of the hexane/acetone (3:2) mixture were added and centrifuged for 10 min at 3000 rpm. Finally, the absorbance at 503 nm of the resulting supernatant was determined. The absorbance was used in Equation (4) to determine the content of lycopene as mg 100 g^−1^ fresh weight.

(4) $$\mathrm{Lycopene}={\mathrm{Abs}}_{503}\times 31.2$$

### 3.18. Vitamin C

This was determined by the titration method with 2,6 dichlorophenolindofenol [51]. A total of 10 g of fresh fruit was weighed and macerated in a mortar with 10 mL of 2% HCl, and it was then filtered through a sterile absorbent gauze into a 100 mL volumetric flask. A 10 mL aliquot was taken and titrated with 2,6-dichlorophenolindofenol until a persistent rosacea coloration was obtained. The results were expressed as mg 100 g^−1^ fresh weight.

### 3.19. Mineral Content

The concentrations of K, Ca, Mg, Na, Fe, Zn, and Cu were measured. For digestion, 0.2 g of dry sample were weighed and 30 mL of concentrated HNO_3_ were added to a 100 mL beaker. It was covered with a watch glass and heated on a grill until complete disintegration of the organic matter (approximately 4 h). The volume of HNO_3_ was topped up several times to avoid drying the sample. When the solution was completely clear (no residue), it was allowed to cool. After this, the solution was filtered on Whatman No. 42 filter paper and taken up to a volume of 50 mL using deionized water in a volumetric flask. Subsequently, dilutions were made in a 1:10 ratio. The concentrations of each element were read in a plasma emission spectrophotometer (ICP, Termo Jarrel Ash ASH model 7400). The total N content in leaves and fruits was determined by the micro-kjeldahl method [52]. For this method, 0.05 g of the dry sample were weighed into a digestion flask, and 4 mL of digest mixture (25 g of K_2_SO_4_, 10 g of red mercury oxide, 1 L of concentrated H_2_SO_4_, and 25 mL of Cu_2_SO_4_) were then weighed and digested (~2 h) in a microdigestor. Subsequently, it was placed in the micro-kjeldahl distiller with 50% NaOH added, and the distillation was then recovered with 30 mL of 2.2% boric acid and 3–5 drops of bromocresol green/methyl red mixed indicator. The titration was carried out with 0.025 N H_2_SO_4_.

### 3.20. Statistical Analysis

The experimental design used was completely randomized with 16 replicates per treatment, with one plant considered as an experimental unit. For the variables of photosynthetic pigments, enzymatic activity, antioxidant capacity, and mineral content, five replicates were used per treatment. The statistical language R CRAN was used, in which an analysis of variance and Fisher Least Significant Difference (α ≤ 0.05) test were performed for all variables.

## 4. Conclusions

The present work shows that the application of both chitosan–PVA and chitosan–PVA + Cu NPs promoted the vegetative and reproductive growth of tomato plants without saline stress. Under saline stress, the application of chitosan–PVA + Cu NPs significantly increased the yield.

The application of chitosan–PVA increased all of the pigments in the leaves, chlorophylls a and b, total chlorophylls, carotenoids, and the activity of superoxide dismutase. On the other hand, the application of chitosan–PVA + Cu NPs increased the activity of all enzymes, namely, ascorbate peroxidase, catalase, superoxide dismutase, glutathione peroxidase, and phenylalanine ammonium lipase, when the plants grew without saline stress. This demonstrates that chitosan–PVA + Cu NPs activate the enzymatic defense mechanism of tomato plants.

The application of chitosan–PVA increased the vitamin C content in tomato fruits, both with and without saline stress. In contrast, the lycopene content on tomato fruits was increased by chitosan–PVA + Cu NPs both with and without saline stress. Therefore, the application of chitosan–PVA and chitosan–PVA + Cu NPs is an alternative for increasing the quality of tomato fruit.

Regarding mineral content, the application of chitosan–PVA decreased Na accumulation in the leaves compared to the NaCl treatment. Therefore, it can be used in tomato plants to induce tolerance to saline stress.

## Figures and Tables

**Table 1:** Parameters of growth and development of tomato plants.

Stress	Treatment	Plant Height (cm)	Stem Diameter (mm)	NFH	FW Shoot (g)	FW Root (g)	DW Shoot (g)	DW Root (g)	Yield (g)
Without Stress	T0	208 a	14.4 b	25.4 b	2582 a	74 b	323 a	14.6 b	5607 a
	CsPVA	200 b	14.9 ab	27.1 ab	2592 a	128 a	342 a	19.6 a	5438 a
	NPsCu	201 b	15.4 a	30.5 a	2551 a	143 a	342 a	20.8 a	5352 a
NaCl	NaCl	148 ab	12.5 a	25.2 a	1087 a	111 a	156 a	18.2 a	888 ab
	Cs–NaCl	151 a	12.7 a	24.6 a	1148 a	131 a	153 a	16.6 a	879 b
	NPsCu–NaCl	144 b	12.5 a	25.5 a	1157 a	128 a	165 a	17.6 a	973 a

T0: Absolute control. CsPVA: 1 g chitosan–PVA hydrogel. NPsCu: 10 mg Cu NPs + chitosan–PVA. NaCl: witness + 100 mM NaCl. Cs–NaCl: Cs–PVA + 100 mM NaCl. NPsCu–NaCl: 10 mg Cu NPs + chitosan–PVA + 100 mM NaCl. NFH: Number of fruits harvested. FW: Fresh weight. DW: Dry weight. Means with the same letter within the same column of each treatment are not different according to Fisher Least Significant Difference test (α ≤ 0.05).

**Table 2:** Content of pigments on tomato leaves (mg g^−1^ fresh weight).

Stress	Treatment	Chlorophyll a	Chlorophyll b	Total Chlorophyll	Chlorophyll a/b	Total Carotenoids
Without Stress	T0	1.71 a	0.52 a	2.23 a	3.31 a	0.29 a
	CsPVA	1.71 a	0.54 a	2.25 a	3.17 ab	0.28 a
	NCu	1.55 a	0.49 a	2.05 a	3.16 b	0.26 a
NaCl	NaCl	2.38 ab	0.76 b	3.14 ab	3.12 a	0.45 ab
	Cs–NaCl	2.74 a	0.93 a	3.67 a	2.94 b	0.52 a
	nCu–NaCl	2.06 b	0.67 b	2.74 b	3.05 ab	0.38 b

T0: Absolute control. CsPVA: 1 g chitosan–PVA hydrogel. NPsCu: 10 mg Cu NPs + chitosan–PVA. NaCl: witness + 100 mM NaCl. Cs–NaCl: Cs–PVA + 100 mM NaCl. NPsCu–NaCl: 10 mg Cu NPs + chitosan–PVA + 100 mM NaCl. Means with the same letter within the same column of each treatment are not different according to Fisher Least Significant Difference test (α ≤ 0.05).

**Table 3:** Enzymatic activity in tomato leaves and fruits.

Organ	Stress	Treatment	APX	CAT	SOD	GPX	PAL
Leaves	Without stress	T0	215.31 b	12.93 b	13.62 b	17.40 b	2.82 b
		CsPVA	203.74 b	13.36 b	19.98 ab	16.46 b	2.67 b
		NCu	337.62 a	27.28 a	26.13 a	24.23 a	4.57 a
	NaCl	NaCl	335.18 a	19.97 a	22.51 b	19.07 a	3.60 a
		Cs–NaCl	354.28 a	18.77 a	29.60 a	21.38 a	4.20 a
		nCu–NaCl	316.18 a	13.84 a	27.04 ab	20.64 a	3.58 a
Fruits	Without stress	T0	445.65 a	26.27 b	12.35 ab	26.49 b	nd
		CsPVA	430.50 a	32.77 b	7.75 b	34.98 a	nd
		NCu	476.83 a	58.38 a	17.32 a	33.11 a	nd
	NaCl	NaCl	566.63 a	98.83 a	48.98 a	33.12 a	nd
		Cs–NaCl	454.20 a	42.47 b	29.17 b	27.95 a	nd
		nCu–NaCl	485.24 a	40.59 b	35.66 ab	28.55 a	nd

T0: Absolute control. CsPVA: 1 g chitosan–PVA hydrogel. NPsCu: 10 mg Cu NPs + chitosan–PVA. NaCl: witness + 100 mM NaCl. Cs–NaCl: Cs–PVA + 100 mM NaCl. NPsCu–NaCl: 10 mg Cu NPs + chitosan–PVA + 100 mM NaCl. APX: Ascorbate peroxidase (μmol of ascorbate min^−1^ by total proteins [mg g^−1^]). CAT: Catalase (mM H_2_O_2_ min^−1^ per total protein [mg g^−1^]). SOD: Superoxide dismutase (U mg^−1^ protein). GPX: Glutathione peroxidase (μmol of glutathione per min by total proteins [mg g^−1^]). PAL: Phenylalanine ammonium lyase (Production of 1 mM cinnamic acid per min by total proteins [mg g^−1^]). Means with the same letter within the same column of each treatment are not different according to Fisher Least Significant Difference test (α ≤ 0.05).

**Table 4:** Antioxidant capacity in tomato leaves.

Stress	Treatment	Antioxidant Capacity Equivalent to Trolox		Antioxidant Capacity Equivalent to Vitamin C		Reduced Glutathione (μmol of GSH per mg of Total Proteins)	Total Phenols (mg of Gallic Acid per g of Fresh Weight)
		ABTS	DPPH	ABTS	DPPH		
Without stress	T0	80.02 a	47.89 a	10.10 a	15.10 a	282.92 b	1.72 b
	CsPVA	58.93 a	56.19 a	7.20 a	17.31 a	286.95 b	2.76 a
	NCu	89.38 a	51.97 a	11.39 a	16.19 a	447.58 a	2.64 a
NaCl	NaCl	91.41 a	49.51 a	11.67 a	15.53 a	221.30 a	2.77 a
	Cs–NaCl	91.74 a	23.48 b	11.71 a	8.62 b	284.00 a	4.27 a
	nCu–NaCl	99.52 a	21.16 b	12.78 a	8.01 b	280.61 a	3.18 a

T0: Absolute control. CsPVA: 1 g chitosan–PVA hydrogel. NPsCu: 10 mg Cu NPs + chitosan–PVA. NaCl: witness + 100 mM NaCl. Cs–NaCl: Cs–PVA + 100 mM NaCl. NPsCu–NaCl: 10 mg Cu NPs + chitosan–PVA + 100 mM NaCl. ABTS: 2,20-Azinobis-3-ethylbenzotiazoline-6-sulphonic acid. DPPH: 2,2-Diphenyl-1-Picrylhydrazyl. Means with the same letter within the same column of each treatment are not different according to Fisher Least Significant Difference test (α ≤ 0.05).

**Table 5:** Antioxidant capacity in tomato fruits.

Stress	Treatment	Antioxidant Capacity Equivalent to Trolox		Antioxidant Capacity Equivalent to Vitamin C		Reduced Glutathione (μmol of GSH per mg of Total Proteins)	Vitamin C (mg 100 g^−1^ Fresh Weight)	Lycopene (mg 100 g^−1^ Fresh Weight)
		DPPH	ABTS	DPPH	ABTS			
Without Stress	T0	68.97 ab	10.96 a	8.58 ab	5.30 a	324.87 b	8.10 b	2.85 b
	CsPVA	54.09 b	17.65 a	6.53 b	7.07 a	431.13 a	11.97 a	3.09 b
	NCu	87.12 a	18.91 a	11.08 a	7.41 a	322.36 b	11.44 a	5.05 a
NaCl	NaCl	83.17 ab	20.24 a	10.53 ab	7.77 a	491.82 a	10.38 b	2.87 a
	Cs–NaCl	99.52 a	30.77 a	12.78 a	8.74 a	282.81 b	14.78 a	2.99 a
	nCu–NaCl	67.39 b	22.78 a	8.36 b	8.44 a	301.00 b	13.90 a	4.07 a

T0: Absolute control. CsPVA: 1 g chitosan–PVA hydrogel. NPsCu: 10 mg Cu NPs + chitosan–PVA. NaCl: witness + 100 mM NaCl. Cs–NaCl: Cs–PVA + 100 mM NaCl. NPsCu–NaCl: 10 mg Cu NPs + chitosan–PVA + 100 mM NaCl. ABTS: 2,20-Azinobis-3-ethylbenzotiazoline-6-sulphonic acid. DPPH: 2,2-Diphenyl-1-Picrylhydrazyl. Means with the same letter within the same column of each treatment are not different according to Fisher Least Significant Difference test (α ≤ 0.05).

**Table 6:** Mineral content in tomato leaves and fruits.

Organ	Stress	Treatment	N (mg g^−1^ DW)	K (mg g^−1^ DW)	Ca (mg g^−1^ DW)	Mg (mg g^−1^ DW)	Na (mg g^−1^ DW)	Fe (µg g^−1^ DW)	Zn (µg g^−1^ DW)	Cu (µg g^−1^ DW)
Leaves	Without Stress	T0	32.20 a	10.61 a	18.50 a	3.07 a	6.33 a	96.8 a	17.6 a	100.8 a
		CsPVA	28.16 a	9.07 b	16.99 b	2.76 a	6.57 a	80.8 a	14.8 a	93.6 b
		NCu	34.12 a	11.21 a	18.92 a	2.00 a	4.59 a	64.6 a	10.8 a	93.8 b
	NaCl	NaCl	21.86 b	6.48 a	14.20 a	5.25 a	104.75 a	91.2 a	32.0 a	123.8 a
		Cs–NaCl	23.13 b	7.14 a	15.05 a	3.58 a	49.82 b	75.0 b	23.0 a	97.0 a
		nCu–NaCl	24.76 a	7.41 a	14.78 a	4.74 a	64.37 ab	79.8 b	21.2 a	97.0 a
Fruits	Without Stress	T0	23.14 b	9.16 a	6.34 a	2.31 a	0.98 a	13.8 a	8.8 a	93.8 a
		CsPVA	24.02 ab	9.42 a	5.21 a	2.24 b	1.88 a	22.4 a	8.4 a	111.8 a
		NCu	26.57 a	9.38 a	7.45 a	2.21 b	1.08 a	15.6 a	7.8 a	92.8 a
	NaCl	NaCl	21.78 a	8.31 a	2.92 a	2.34 a	4.30 a	13.8 a	11.0 a	93.2 a
		Cs–NaCl	22.39 a	8.39 a	2.83 a	2.32 a	3.85 a	13.8 a	8.8 a	93.4 a
		nCu–NaCl	23.20 a	8.89 a	2.50 a	2.33 a	4.42 a	10.4 a	10.6 a	93.0 a

T0: Absolute control. CsPVA: 1 g chitosan–PVA hydrogel. NPsCu: 10 mg Cu NPs + chitosan–PVA. NaCl: witness + 100 mM NaCl. Cs–NaCl: Cs–PVA + 100 mM NaCl. NPsCu–NaCl: 10 mg Cu NPs + chitosan–PVA + 100 mM NaCl. DW: Dry weight. Means with the same letter within the same column of each treatment are not different according to Fisher Least Significant Difference test (α ≤ 0.05).

## Supplementary Materials

The Supplementary Materials are available online.
