# Supplementary material for: Effects of Chitosan–PVA and Cu Nanoparticles on the Growth and Antioxidant Capacity of Tomato under Saline Stress
Source: Molecules. 2018 Jan 16;23(1):178. doi: 10.3390/molecules23010178 (PMC6017526; doi:10.3390/molecules23010178)
Supplement: Supplementary file 1 [file molecules-23-00178-s001.pdf]

Supplementary Materials

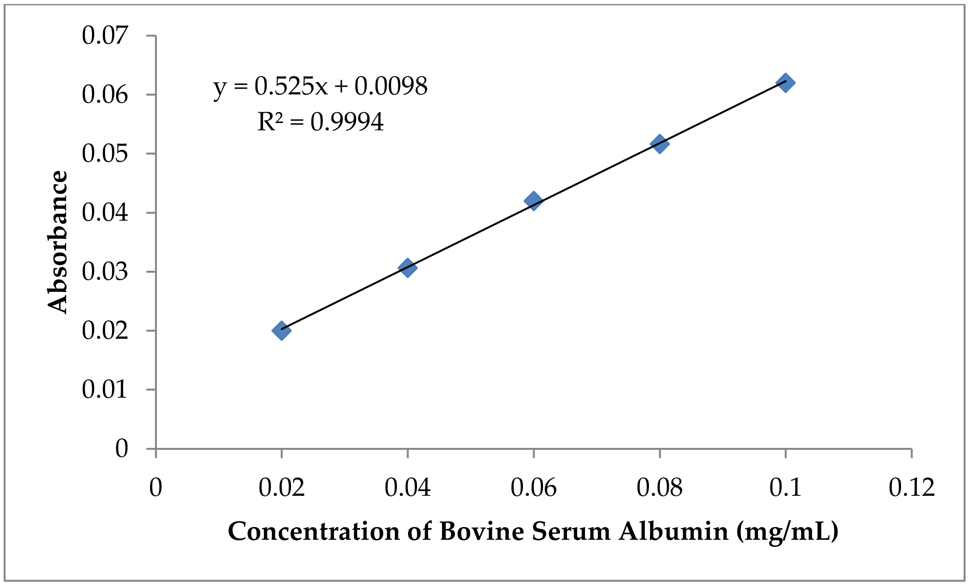

Figure S1. Calibration curve obtained for the quantification of proteins.

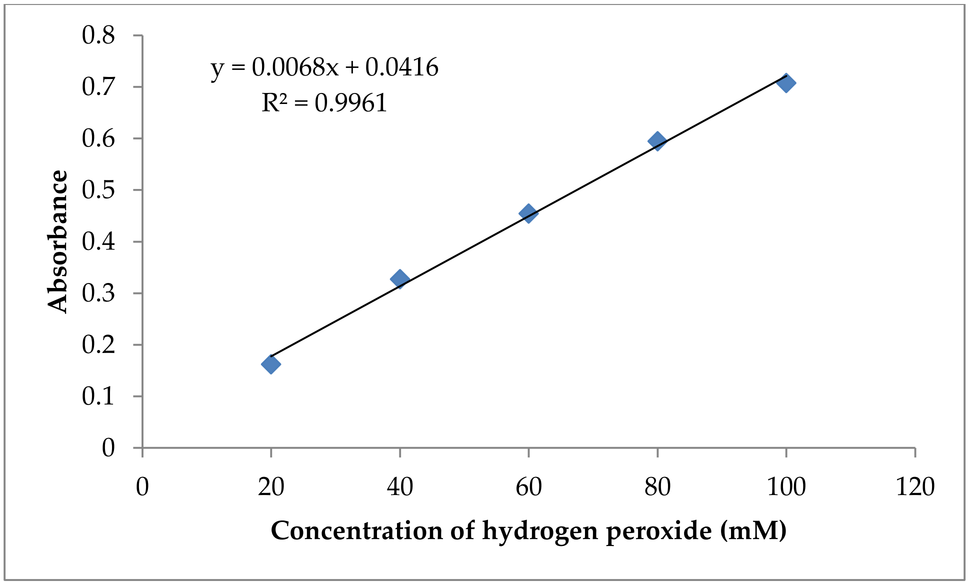

Figure S2. Calibration curve obtained for the quantification of hydrogen peroxide to determine Catalase.

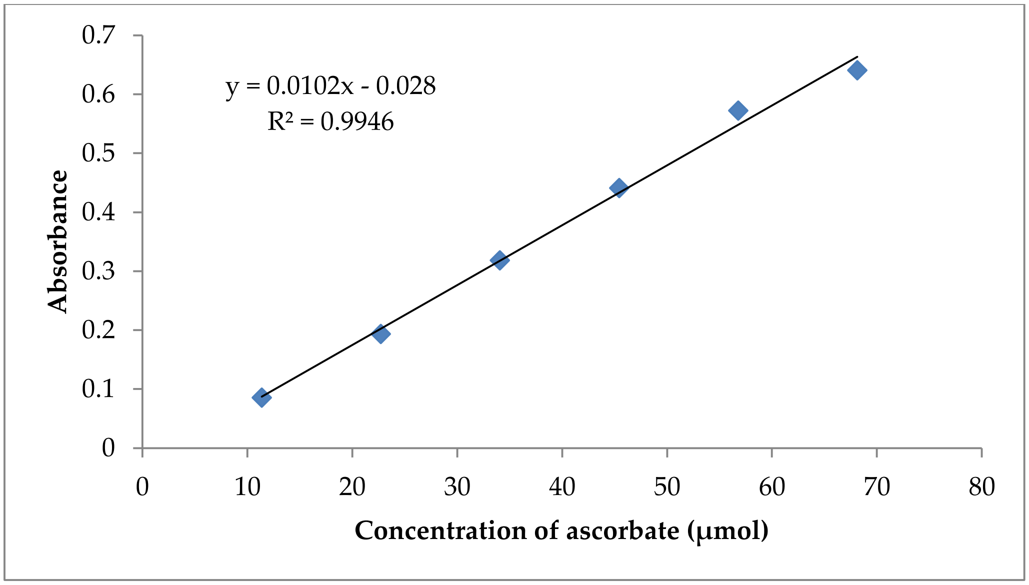

Figure S3. Calibration curve obtained for the quantification of ascorbate to determine APX.

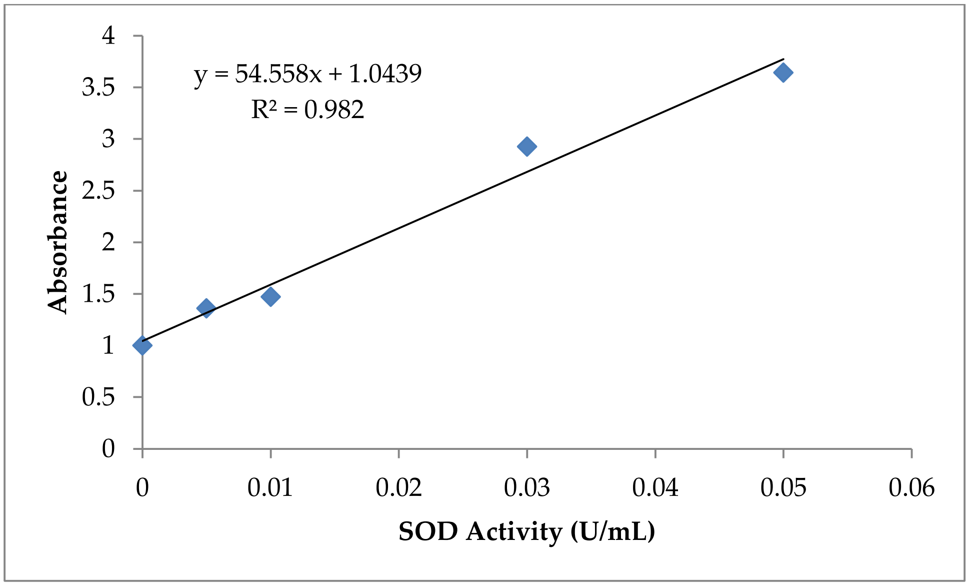

Figure S4. Calibration curve obtained to calculate the activity of the SOD.

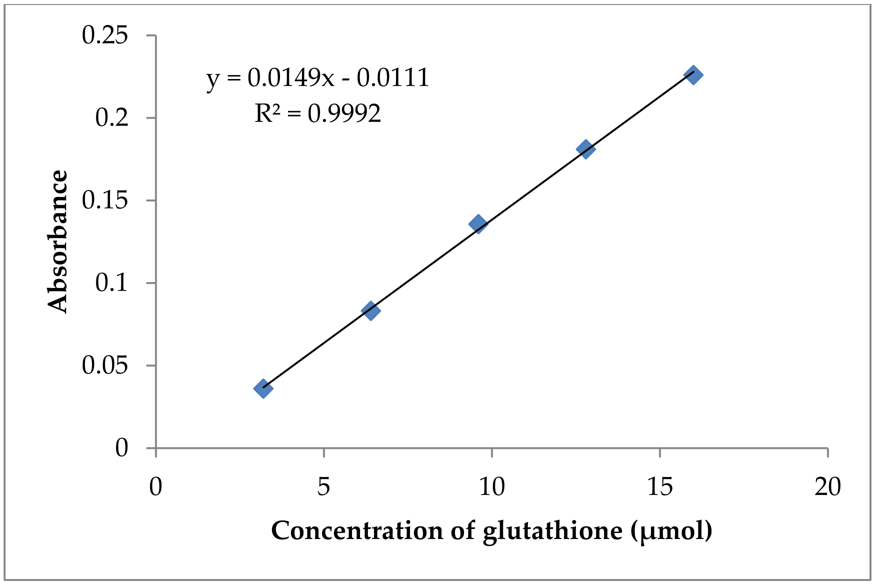

Figure S5. Calibration curve obtained for the quantification of glutathione to determine GPX y GSH.

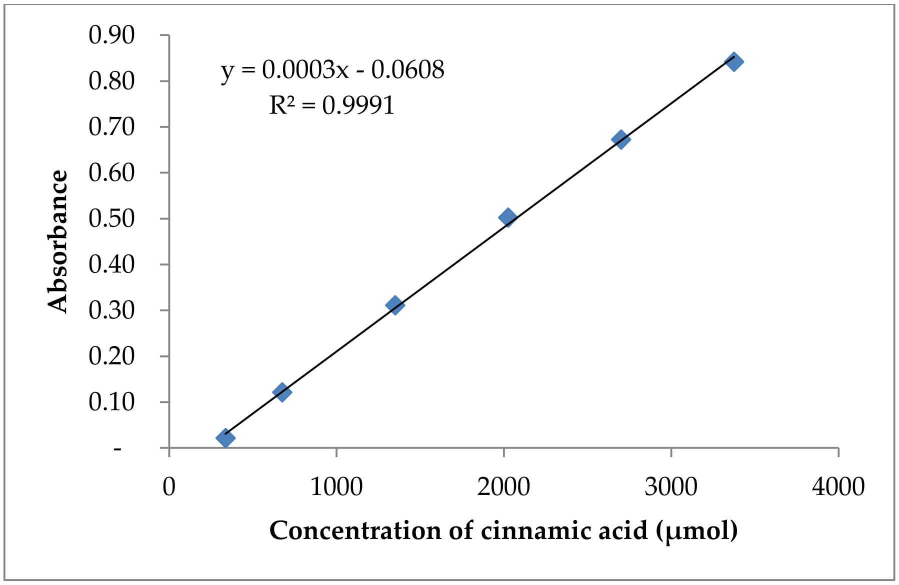

Figure S6. Calibration curve obtained for the quantification of cinnamic acid to determine PAL.

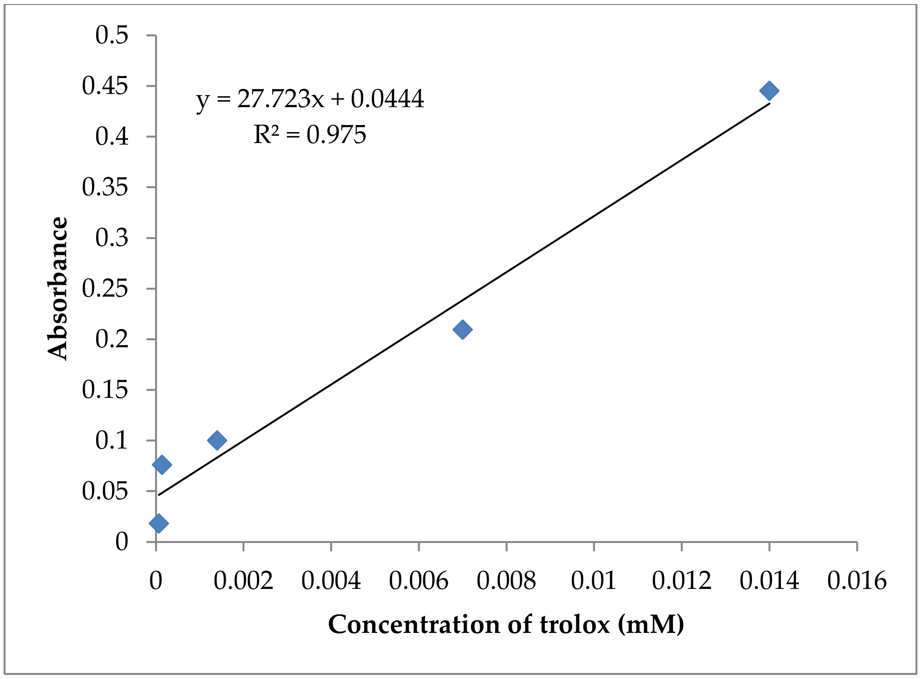

Figure S7. Calibration curve obtained for the quantification of trolox by the ABTS technique.

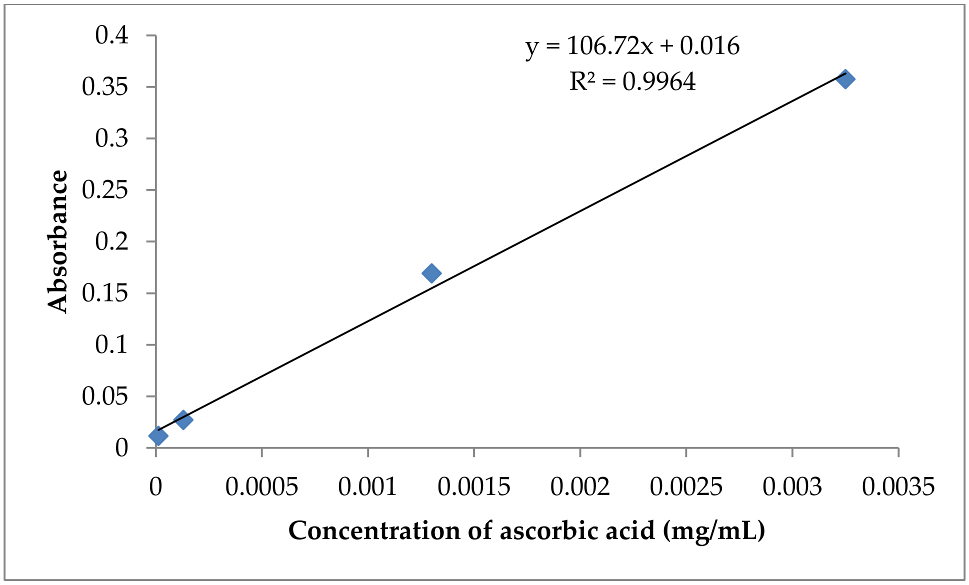

Figure S8. Calibration curve obtained for the quantification of ascorbic acid by the ABTS technique.

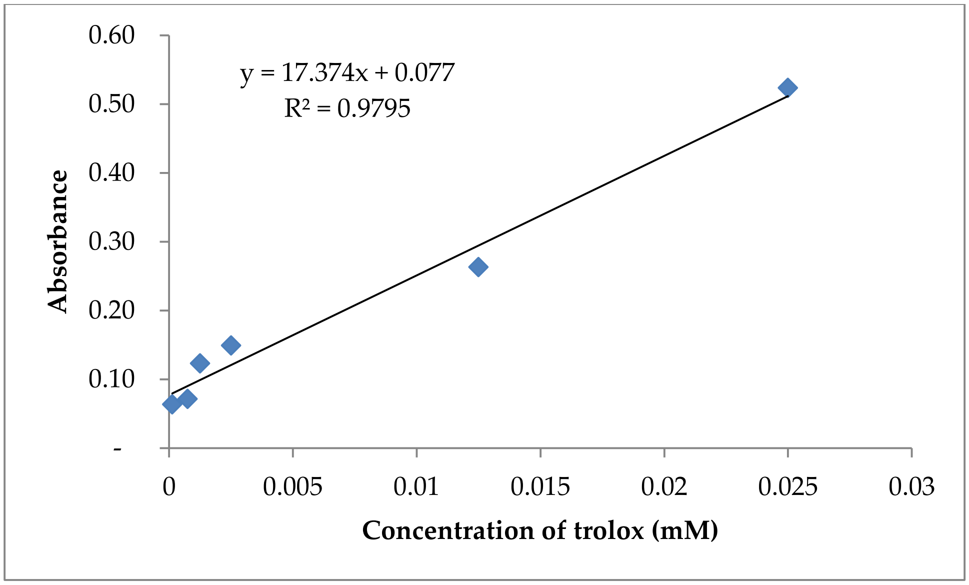

Figure S9. Calibration curve obtained for the quantification of trolox by the DPPH technique.

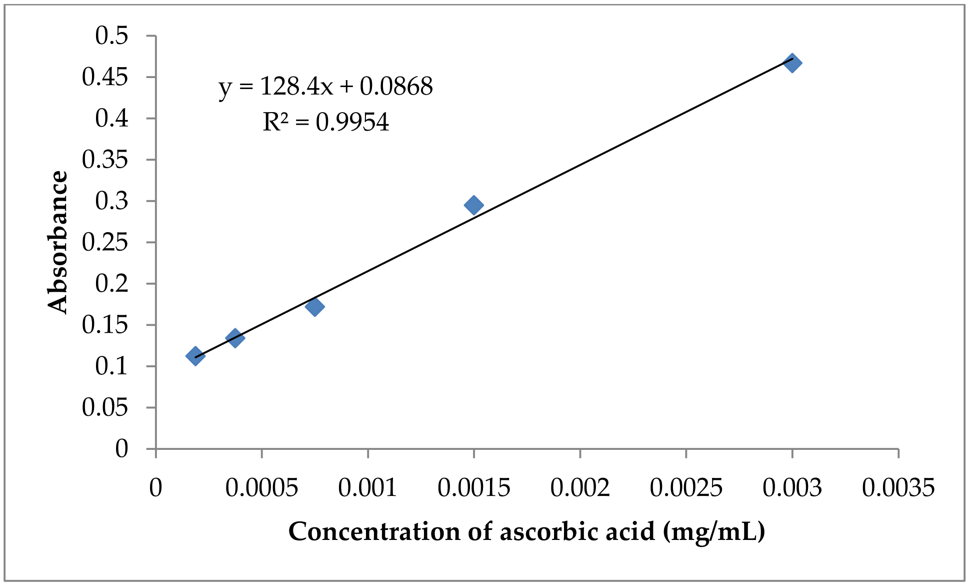

Figure S10. Calibration curve obtained for the quantification of ascorbic acid by the DPPH technique.

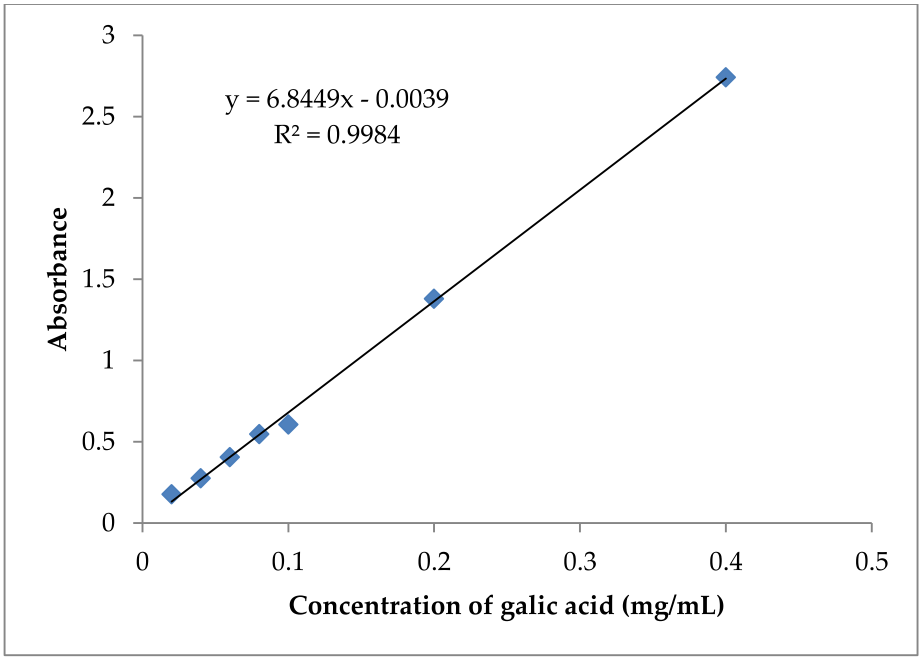

Figure S11. Calibration curve obtained for the quantification of galic acid to determine total phenols.
